# Ashkenazi Jewish and Other White *APC* I1307K Carriers Are at Higher Risk for Multiple Cancers

**DOI:** 10.3390/cancers14235875

**Published:** 2022-11-29

**Authors:** Esther Forkosh, Michael Bergel, Kathryn E. Hatchell, Sarah M. Nielsen, Brandie Heald, Ariel A. Benson, Eitan Friedman, Edward D. Esplin, Lior H. Katz

**Affiliations:** 1 Department of Gastroenterology, Hadassah Medical Center, Jerusalem 91120, Israel; 2 Faculty of Medicine, Hebrew University of Jerusalem, Jerusalem 91121, Israel; 3 Medical Affairs, Invitae, San Francisco, CA 94103, USA; 4 The Meirav Center High Risk Clinic, Sheba Medical Center, Ramat-Gan 52621, Israel

**Keywords:** *APC* I1307K, cancer, Ashkenazi jews, CRC

## Abstract

**Simple Summary:**

*APC *I1307K has a two-fold increased risk for colorectal cancer in Ashkenazi Jews (AJ) compared to non-Jewish populations. The study aims to demonstrate the prevalence of the APC I1307K variant in the largest cohort of AJ and non-AJ white (NAW) descents described so far. In addition, we assessed the prevalence of CRC and extracolonic malignancies among I1307K carriers. We found that NAW I1307K carriers had a higher risk of any cancer, such as CRC, melanoma, breast, and prostate cancer. Among AJ, the variant increased the risk for CRC and renal cancer, and AJ men had a higher risk for any cancer and melanoma. We believe these findings are significant and may suggest the necessity for cancer screening in this population.

**Abstract:**

Purpose: *APC* I1307K has a higher prevalence among Ashkenazi Jews (AJ), and a two-fold increased risk for colorectal cancer (CRC) compared to non-Jewish populations. We assessed CRC and extracolonic malignancies among I1307K carriers from AJ and non-AJ whites (NAW). Methods: We compared the rate of I1307K in cancer patients who underwent germline genetic testing via a multi-gene panel with healthy subjects retrieved from the gnomAD database. Cases undergoing testing were not selected and testing was undertaken through a commercial laboratory. Results: Overall, 586/7624 (7.6%) AJ with cancer carried I1307K compared to 342/4918 (6.9%) in the AJ control group (*p* = NS). In the NAW, 318/141,673 (0.2%) cancer patients and 73/58,918 (0.1%) controls carried the variant [OR = 1.8, (95% CI 1.41–2.35), *p* < 0.001]. I1307K in NAW was associated with an increased risk of CRC [OR = 1.95, (95% CI 1.39–2.73), *p* < 0.01], melanoma [OR = 2.54, (95% CI 1.57–3.98)], breast [females, OR = 1.73, (95% CI 1.18–2.65), *p* < 0.01], and prostate cancer [males, OR = 2.42, (95% CI 1.45–3.94), *p* < 0.01]. Among AJ, the variant increased the risk for CRC [OR = 1.67, (95% CI 1.36–2.05), *p* < 0.001] and renal cancer [OR = 1.64, (95% CI 1.04–2.47)]. AJ men had a higher risk for any cancer [OR = 1.32, (95% CI 1.05–1.66), *p* < 0.05] and melanoma [OR = 2.04, (95% CI 1.24–3.22); *p* < 0.05]. Conclusions: This is the most extensive study to date conducted on I1307K carriers, although it is amenable to selection bias. NAW carrying I1307K had a higher risk of any cancer and several specific cancer types, whereas AJ carrying the variant had a risk for only a few select cancers. Our data add to the research base on I1307 carriers concerning future risk management.

## 1. Introduction

Colorectal cancer (CRC) is the third most common cancer and is also the third leading cause of cancer-related mortality in the world [1,2,3]. CRC cases are usually sporadic (i.e., no discernible inherited contribution), but are hereditary in up to 15% of cases [4,5,6]. Hereditary CRC is, for the most part, accounted for by the Lynch syndrome (OMIM# 120435), familial adenomatous polyposis (FAP) (OMIM# 175100), and *MUTYH*-associated polyposis (MAP) (OMIM# 608456) [7,8]. Ashkenazi Jews (AJ) are at the highest risk for developing CRC compared with all other Israeli ethnic groups. This elevated risk is similar to that of an individual with a positive family history and higher than the 5–6% lifetime risk for the general Western population [5].

The adenomatous polyposis coli (*APC*) gene is an important tumor suppressor gene that encodes for a key regulatory protein of the Wnt -CTNNB1(β-catenin) signaling pathway [9,10,11]. Somatic genetic alterations along this pathway are noted in the majority of CRC cases. In fact, the inactivation of the *APC* gene by somatic biallelic variants contributes to colorectal tumorigenesis in more than two-thirds of sporadic CRC cases [12]. In FAP cases, a germline heterozygous pathogenic loss of function variant in *APC* is present. When a somatic pathogenic variant inactivates the wild-type parental allele, the lack of a functional APC protein results in the formation of hundreds to thousands of small adenomas, one or more of which will eventually undergo a malignant transformation [13].

In 1997, Laken et al. identified a missense variant in codon 1307 within the *APC *gene. This variant, the transversion of T to A at nucleotide 3920, changes the sequence from A3TA4 to A8, resulting in a genetically unstable hypermutable region of DNA that is prone to somatic mutations [14,15]. According to one meta-analysis (HuGE Review and Meta-analysis) [16], *APC* I1307K has a higher prevalence among AJ (pooled prevalence = 11.8%) than in non-Ashkenazi Jews (pooled prevalence = 2.9%) or the non-Jewish population (pooled prevalence = 0.92%) [16]. Among AJ, *APC* I1307K is associated with a pooled odds ratio (OR) of 2.17 for CRC [16]. It is unclear whether *APC* I1307K is related to a higher risk for adenoma formation or the conversion from benign polyps to malignant polyps; however, compared to FAP, *APC* I1307K has lower penetrance, and the protein remains functional [17].

Several studies have reported the possible risks attributable to *APC* I1307K and extracolonic tumors such as prostate, skin, ovarian, and breast, with inconclusive results [13,18,19,20]. The association of extracolonic malignancies among non-Jewish *APC* I1307K carriers has not been well studied. The purpose of this study, based on a large genetic testing dataset, is to assess the risk of CRC and extracolonic malignancies among AJ as well as non-AJ whites (NAW) *APC* I1307K carriers.

## 2. Methods

### 2.1. Study Participants

Subjects with a reported history of cancer who underwent germline genetic testing at a single commercial laboratory (Invitae, San Francisco, CA, USA) that included the *APC *gene between October 2015 and March 2020 were included. All patient data were de-identified before analysis under the Western Institutional Review Board (IRB) protocol number 1167406. Due to the relatively small sample size of ethnicities other than AJ and NAW, we included only individuals from these two ethnicities in this study. The NAW group included participants who self-reported French Canadian or white ethnicity. Exclusion criteria were: (1) subjects without cancer; (2) subjects carrying pathogenic or likely pathogenic variants other than *APC* I1307K; and (3) subjects who were not AJ or NAW or were reported as mixed AJ or mixed NAW.

### 2.2. Data Query and Analysis

Data were extracted from the test requisition form, completed by the ordering healthcare provider, and included clinical information regarding patients’ country of residence, self-reported ethnicity, age at the time of genetic testing, and type of cancer, when available. Only cancer types that had a minimum of 20 patients in each group were included in the statistical analysis to ensure meaningful statistical results. Cancer types with less than 20 patients are mentioned without analysis. The control group was acquired from gnomAD v2.1.1 [21] (non-cancer) and included individuals who participated in cancer studies and did not have a malignancy. Ethnicity was determined by an “unambiguous cluster” with the major population in principal component analysis.

### 2.3. Genetic Testing 

Sample types for this cohort included germline DNA extracted from blood or saliva. Once extracted, DNA was processed and subjected to paired-end sequencing on an Illumina next-generation sequencing platform (depth of coverage 50× minimum, 350× average) [22]. Variants were subjected to clinical interpretation using the refined American College of Medical Genetics and Genomics criteria [23].

### 2.4. Statistical Analysis 

We performed Statistical analyses using R. We assessed differences in variant frequency between groups with a two-sided test using the Chi-square test function (https://www.rdocumentation.org/packages/stats/versions/3.6.2, accessed on 19 February 2022). Additionally, we used a test of a Chi-square distribution and applied Yate’s correction for continuity for small sample sizes when necessary. OR was calculated by using the odds ratio function (Tomas J. Aragon (2020). epitools: Epidemiology Tools. R package version 0.5–10.1. https://CRAN.R-project.org/package=epitools, accessed on 19 February 2022). Statistical significance was defined as *p* < 0.05 after multiple testing corrections.

## 3. Results

### 3.1. Participant Characteristics

**Figure 1:**
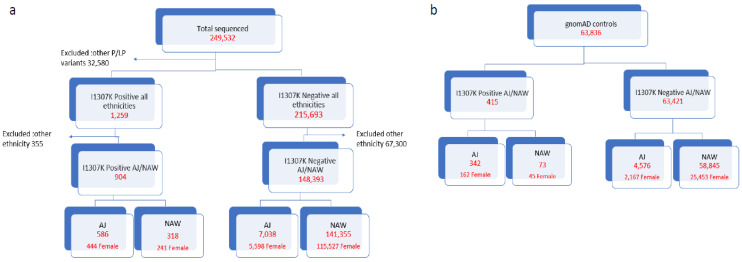
(**a**) Study group (cancer patients); (**b**) control group (individuals from gnomAD 2.1—non-cancer).

Overall, 249,532 individuals with any type of cancer underwent multigene panel testing (MGPT) with a cancer panel that included *APC* (Figure 1a). Of these, 32,580 individuals were excluded since they had another pathogenic/likely pathogenic variant other than APC I1307K (a list of excluded genes and mutations is provided in Table S1). An additional 67,655 individuals were excluded as they were from ethnicities other than AJ and NAW. The excluded ethnicities are described in Table S2. The control group was composed of 63,836 NAW and AJ individuals from gnomAD (Figure 1b).

### 3.2. Overall Cancer Rate

**Figure 2:**
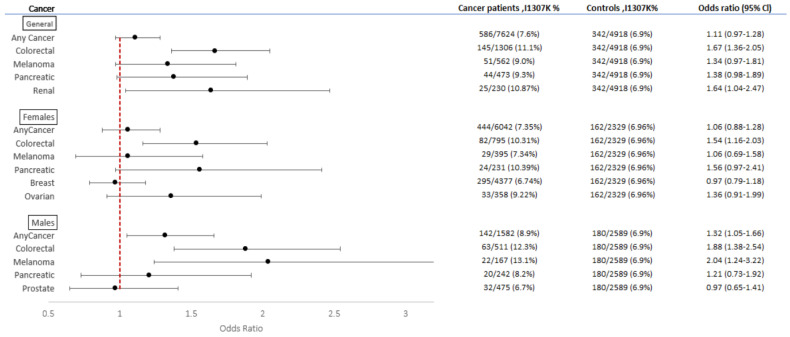
Forest plot representing the OR for different types of cancer according to the *APC* I1307K variant status in AJ.

**Figure 3:**
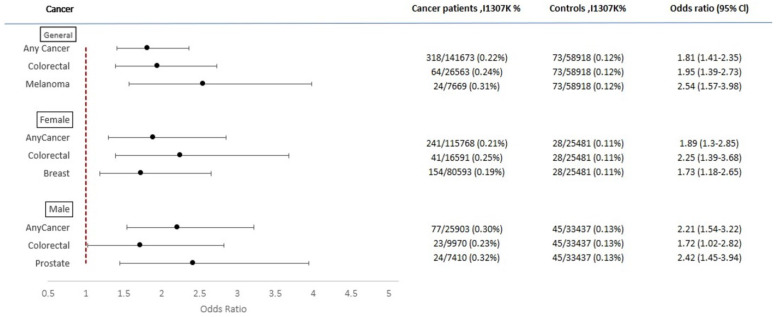
Forest plot representing the OR for different types of cancer according to the *APC* I1307K variant status in NAW.

Overall, 586/7624 (7.6%) AJ with any cancer carried the I1307K *APC* variant compared to 342/4918 (6.9%) in the AJ control group (*p* = NS) (Figure 2); however, among AJ males with any cancer, the rate of I1307K *APC* was significantly higher than in AJ males without cancer [OR 1.32 (95% CI 1.05–1.66), *p* < 0.05]. In the NAW group, 318/141,673 (0.2%) participants with cancer were carriers of I1307K *APC* while only 73/58,918 (0.1%) individuals in the control group carried it [OR 1.81 (95% CI 1.41–2.35), *p* < 0.001]. These results were similar after stratification by sex (Figure 3). The results demonstrate a significantly higher *APC* I1307K variant prevalence among AJ than NAW in both cancer patients and healthy individuals (*p* < 0.001 for both groups).

### 3.3. CRC

*APC* I1307K was more prevalent among CRC patients in both ethnicities as compared to healthy individuals [OR 1.67 (95% CI 1.36–2.05), *p* < 0.001 for AJ (Figure 2) and OR 1.95 (95% CI 1.39–2.73), *p* < 0.01 for NAW (Figure 3)]. Statistical significance was maintained in both groups after sex stratification as well.

### 3.4. Other Types of Cancers

We assessed the rate of the *APC* I1307K variant in patients with different types of cancer and at least 20 cases of the same cancer type. Among AJ, renal cancer (for either sex) and melanoma (in males only) were noted at a higher prevalence among *APC* I1307K carriers compared with healthy individuals [OR 1.64 (95% CI 1.04–2.47) and OR 2.04 (95% CI 1.24–3.22); *p* < 0.05, respectively (Figure 2)]. The rates of the *APC* I1307K variant amongst breast (females only), ovarian, and pancreatic cancer in AJ were not significantly different compared with healthy controls.

Among NAW, the *APC* I1307K rate was higher among melanoma patients in both sexes [OR 2.54 (95% CI 1.57–3.98), *p* < 0.01 (Figure 3)], as well as in breast (female only) and prostate (male only) cancer patients [OR 1.73 (95% CI 1.18–2.65), *p* < 0.01; OR 2.42 (95% CI 1.45–3.94), *p* < 0.01, respectively (Figure 3)]. 

In cancer types with smaller sample sizes, the *p*-value was not calculated; however, a trend towards a high rate of I1307K *APC* variant in gastric and lung cancers among AJ, and a high rate of I1307K *APC* in hematologic, lung, ovarian, pancreatic, and renal cancer in the NAW group was observed. Moreover, we observed an even higher rate of urothelial cancer in the NAW, group [OR 4.03 (95% CI 1.78–7.89)]. Differences are specified in Tables S3 and S4.

## 4. Discussion

This study showed that the *APC* I1307K variant is a moderate-risk allele for CRC both in AJ as well as in NAW. Moreover, it seems it may confer a moderately increased risk of developing renal cancer (for either gender of AJ ancestry), and a higher risk of any cancer and melanoma in AJ men. The risk for developing cancers, other than CRC, in AJ *APC* I1307K carriers was previously reported. Woodage and colleagues [13] reported a higher overall cancer risk, excluding non-melanoma skin cancer in AJ carrying the *APC* I1307K variant (OR 1.5, 95% CI 1.1–2.0, *p* = 0.01) and a higher frequency of breast cancer in first-degree relatives (OR 1.4, 95% CI 1.1–1.8, *p* = 0.01). Leshno and colleagues [20] showed an increased risk for any cancer and several specific types of cancer such as pancreas (OR 3.71, 95% CI 1.71–8.03, *p* < 0.001), lung (OR 7.3, 95% CI 2.58–20.7, *p* < 0.0001), urinary tract (OR 4.5, 95% CI 1.49–13.57, *p* < 0.001), and skin (OR 3.25, 95% CI 1.44–7.36, *p* < 0.001) in men and breast (OR 2.84 95% CI 1.74–4.66, *p* < 0.0001) and skin cancer (OR 4.81, 95% CI 2.90–7.97, *p* < 0.0001) in women in an Israeli-based study. One possible explanation to account for the differences between these studies and the current study is the exclusion of other pathogenic variants in the study reported herein, an exclusion not reported to be implemented in these past studies. By not excluding other pathogenic variants associated with hereditary cancer risk, the actual assessment of the risk conferred solely by the *APC* I1307K variant may have been overestimated by the presence of other pathogenic variants that confer a substantially increased risk common in AJ (e.g., *BRCA1* and *BRCA2*). Furthermore, these past studies encompassed much smaller analyzed case cohorts (Leshno 14,624; Woode 5081) compared to the larger dataset reported here. 

Studies from several European countries described the I1307K *APC* variant rate in CRC NAW patients. In cohort studies from Croatia (*n* = 73) [24], Sweden (*n* = 194) [25], and England (*n* = 134) [26] among the NAW population, none carried the variant. Similarly, a study that included 345 African Americans, Italians, Finns, and Hawaiian–Japanese did not find any I1307K *APC* variant carriers [27]. An additional study from Norway found only one I1307K *APC* carrier from amongst 210 patients with CRC and this patient was reported as Jewish [28]. The HuGE review and meta-analysis [16] reported that the pooled prevalence of I1307K *APC* among non-Jewish CRC cases was 0.92% (95% CI 0.51–1.66), calculated from nine studies. This rate is higher than the 0.24% prevalence in the NAW I1307K *APC* variant carriers in the current study, which is much larger and includes only NAW without mentioning other non-NAW and non-AJ ethnicities. 

Studies conducted on the non-AJ population in Israel found the *APC* I1307K variant at low rates in the Arab population [29]; it was detected in eight families in one study [30] and 2 out of 65 Israeli Arab CRC individuals (2/65, 3.1%) in another study [31]. Moreover, studies focusing on Yemenite Jews (an ethnic group distinct from AJ or Sephardi Jews) reported 3/4 (75%) of CRC patients carried the I1307K *APC* variant, compared to 9/189 (4.7%) healthy carriers in the general Yemenite population [32]. Another study could not detect the variant among 18 Yemenite Jews with CRC [29].

As reported by a Molecular Epidemiology of Colorectal Cancer (MECC) study [33], the *APC* I1307K variant arose in the Middle East, alleles likely existed between 947 BC and 195 BC, at about the time of the beginning of the Jewish diaspora, therefore explaining its presence in the non-Ashkenazi Jewish populations and even in the Arab population in Israel. Its high representation in the AJ population can be explained by genetic drift caused by a specific founder effect in the AJ which happened due to the massive growth of the AJ population in the 18–19 centuries from a small founder population surviving the massacres in Eastern Europe in the late 17 century [33]. Therefore, the low rate of *APC* I1307K observed in NAW may represent either a true incidence in the NAW population or a false inclusion of AJ individuals resulting from incorrect self-reported ancestry due to a lack of data regarding the Jewish origin in the family. 

Our study showed for the first time that in a large population of NAW patients carrying the I1307K *APC* variant, there is an increased risk of overall cancer rate, in addition to an increased risk of CRC, melanoma, breast, and prostate cancer. We also provide data on other malignancies; however, due to the small sample size of each group, it is not possible to determine a statistically significant association. The statistically significant findings from our study regarding the association of *APC* I1307K with cancer in the NAW population may be the result of a large sample size; however, we still feel that the results are clinically significant based on the OR, found to be greater than 1.7, in all comparisons. Although fewer subjects were included in the AJ group, we do not feel that the smaller sample size impacted the analysis since we included more patients and used strict inclusion criteria compared to other studies [1,12,18]. Therefore, we believe that the statistically significant results of the NAW group cannot be extrapolated to the AJ group. 

The only guideline that addresses management recommendations for *APC* I1307K is the National Comprehensive Cancer Network (NCCN). The NCCN recommends that patients with *APC* I1307K undergo a colonoscopy at age 40 with a follow-up every five years [34]. The guidelines add that there is insufficient evidence to determine whether the risk for CRC associated with *APC* I1307K differs among non-AJ individuals and recognize that some individuals may not be aware of AJ heritage. Our data support the association of *APC* I1307K with CRC regardless of ethnicity and variant rate in the population. Yet, the data presented in the current study does not address age at diagnosis and the adequacy of the recommended early detection scheme by the NCCN. We believe that a routine genetic screening test is still not necessary for NAW due to the rarity of the variant. However, based on the results, CRC surveillance by a colonoscopy every five years may be considered in both AJ and NAW I1307*APC* variant carriers and a melanoma checkup should be considered as well.

The limitations of the current study should be acknowledged. First, a selection bias of the individuals who were referred for MGPT, since their referring clinician suspected that there may be an inherited cancer predisposition, based on clinical criteria. Thus, these do not really represent unselected CRC cases or the general population, and cancer risks could have been overestimated due to this [3,35]. Despite this potential bias, the fact that in the present study the OR for developing cancer was lower for both AJ and NAW compared to a previously reported meta-analysis16 is indicative that the reported results were minimally, if at all, affected by any putative selection bias. An additional limitation is that the age of the diagnosis, personal history of colonic polyps, and the cancer family history were not consistently available. These are important factors for assessing the degree of risk [35,36], and accordingly, deciding the appropriate timing for performing the screening. Finally, while the ethnic origin of the individuals in the control group was determined by genetic testing, the study group’s ethnic heritage was self-reported and not independently validated. Thus, to create a more accurate comparison with the control group, we excluded individuals of mixed Ashkenazi descent (when one of the parents is not AJ) from the study group. Furthermore, it cannot be excluded that the higher cancer risk among AJ patients is due to a founder variant in an untested gene, although we are not familiar with such a gene that was not tested in our MGPT. Despite these limitations, the population in our study is one of the most extensively tested, so far, for the presence of the *APC* I1307K variant, in which 586/7624 AJ cancer patients and 318/141,673 NAWs with cancer carry the variant. Our research methodology examined the effect of the variant in isolation from the effect of other known pathogenic variants common in AJ and NAW, hence the results cannot be attributed to other known pathogenic variants other than *APC* I1307K. We used statistical methods, including multiple hypothesis testing correction and correction for small groups as needed, to ensure that our significant results are meaningful.

## 5. Conclusions

Although *APC* I1307K is rare in NAW compared to AJ, it is indeed a moderate cancer risk allele for some cancer types, in addition to CRC. Further research is needed to support our findings in large AJ and NAW general populations and other ethnicities in order to minimize selection bias and to determine whether this variant impacts the age at cancer diagnosis and/or prognosis. These latter factors could determine the clinical recommendations for early detection and risk-reduction schemas.

## Data Availability

Data supporting the reported results can be obtained from the corresponding author.

## Supplementary Materials

The following supporting information can be downloaded at: https://www.mdpi.com/article/10.3390/cancers14235875/s1, Table S1: Genes excluded from the analysis due to any pathogenic/likely pathogenic variant; Table S2: Number of patients from ethnicities excluded from the analysis; Table S3: Patients’ data in cancer types excluded from the analysis; Table S4: Patients’ data in cancer types excluded from the analysis.
