# Supplementary material for: Ashkenazi Jewish and Other White APC I1307K Carriers Are at Higher Risk for Multiple Cancers
Source: Cancers (Basel). 2022 Nov 29;14(23):5875. doi: 10.3390/cancers14235875 (PMC9740723; doi:10.3390/cancers14235875)
Supplement: Supplementary file 1 [file cancers-14-05875-s001.zip › cancers-1969575-supplementary.pdf]

Supplementary Materials:

Ashkenazi Jewish and other White *APC* I1307K Carriers are at Higher Risk for Multiple Cancers

Table S1. Genes excluded from the analysis due to any pathogenic/likely pathogenic variant.

Excluded mutation	
*APC**	*MLH1*
ATM	*MSH6*
BMPR1A	*MUTYH*
BRCA1	*PMS2*
BRCA2	*PALB2*
BRIP1	*PTEN*
CDH1	*SMAD4*
CHECK2	*STK11*
EPCAM	*TP53*
MSH2	*HOXB13* G84E#

* - not including I1307K

\# - only this variant

Table S2. Number of patients from ethnicities excluded from the analysis.

Ethnicities	Carrier	Non-Carrier	Sum of Risk
Asian	8	8946	13
Black/African-American	4	14147	8
Hispanic	38	13629	10
Mediterranean	9	571	0
Mixed	12	7739	30
Native American	0	589	1
Pacific Islander	0	408	1
Sephardic Jewish	8	689	0
Other *	276	20582	56

***Other include other, unkown and AJ-mixed**

Table S3. NAW patients' data in cancer types excluded from the analysis.

NAW	Cancer patients	Control	OR (95% CI)
Brain tumor	2/1315 (0.15%)	73/58918 (0.12%)	1.32 (0.2-4.19)
Colon polyps	26/9819 (0.26%)	73/58918 (0.12%)	2.15 (1.35-3.32)
Endo/Neuroendo	1/1545 (0.06%)	73/58918 (0.12%)	0.6 (0.03-2.65)
Gastric	3/1538 (0.20%)	73/58918 (0.12%)	1.66 (0.39-4.44)
Head neck	1/559 (0.18%)	73/58918 (0.12%)	1.65 (0.07-7.33)
Hematologic	10/2393 (0.42%)	73/58918 (0.12%)	3.43 (1.65-6.35)
Lung	8/2588 (0.31%)	73/58918 (0.12%)	2.54 (1.12-4.98)
Non-Melanoma Skin	1/475 (0.21%)	73/58918 (0.12%)	1.94 (0.08-8.64)
Ovarian	22/9519 (0.23%)	73/58918 (0.12%)	1.88 (1.14-2.97)
Pancreatic	17/7802 (0.22%)	73/58918 (0.12%)	1.77 (1.01-2.94)
Renal	12/3608 (0.33%)	73/58918 (0.12%)	2.72 (1.4-4.83)
Sarcoma	3/1660 (0.18%)	73/58918 (0.12%)	1.53 (0.36-4.11)
Urothelial	8/1637 (0.49%)	73/58918 (0.12%)	4.03 (1.78-7.89)

Table S4. AJ patients' data in cancer types excluded from the analysis.

AJ	Cancer patients	Control	OR (95% CI)
Brain tumor	4/81 (4.94%)	342/4918 (6.95%)	0.72 (0.21-1.75)
Colon polyps	46/424 (10.85%)	342/4918 (6.95%)	1.63 (1.17-2.24)
Endo/Neuroendo	6/83 (7.23%)	342/4918 (6.95%)	1.07 (0.41-2.28)
Gastric	12/118 (10.17%)	342/4918 (6.95%)	1.53 (0.79-2.7)
Head neck	4/30 (13.33%)	342/4918 (6.95%)	2.13 (0.61-5.53)
Hematologic	14/224 (6.25%)	342/4918 (6.95%)	0.9 (0.49-1.51)
Lung	19/197 (9.64%)	342/4918 (6.95%)	1.44 (0.86-2.28)
Non-Melanoma Skin	3/26 (11.54%)	342/4918 (6.95%)	1.82 (0.41-5.31)
Renal	25/230 (10.87%)	342/4918 (6.95%)	1.64 (1.04-2.47)
Sarcoma	8/111 (7.21%)	342/4918 (6.95%)	1.06 (0.47-2.06)
Urothelial	7/116 (6.03%)	342/4918 (6.95%)	0.88 (0.37-1.77)
